# The Netrin-1-Neogenin-1 signaling axis controls neuroblastoma cell migration via integrin-β1 and focal adhesion kinase activation

**DOI:** 10.1080/19336918.2021.1892397

**Published:** 2021-03-16

**Authors:** Andrea A. Villanueva, Pilar Sanchez-Gomez, Ernesto Muñoz-Palma, Sofía Puvogel, Bárbara S. Casas, Cecilia Arriagada, Isaac Peña-Villalobos, Pablo Lois, Manuel Ramírez Orellana, Fabiana Lubieniecki, Fernando Casco Claro, Iván Gallegos, Javier García-Castro, Vicente A. Torres, Verónica Palma

**Affiliations:** a Laboratory of Stem Cells and Developmental Biology, Faculty of Sciences. Universidad de Chile, Santiago, Chile; b Neurooncology Unit, Unidad Funcional de Investigación en Enfermedades Crónicas (UFIEC), Instituto de Salud Carlos III (ISCIII), Madrid, Spain; c Institute for Research in Dental Sciences, Faculty of Dentistry, Universidad de Chile, Olivos 943, Independencia, Santiago, Chile; d Hospital de Pediatría Dr. Prof. Juan P. Garrahan, Buenos Aires, Argentina; e Postgraduate in Education Department, Faculty of Humanities, Universidad Mayor. Santiago, Chile; f Unidad Anatomía Patológica, Unilabs, Madrid, España; g Universidad de Chile, Universidad de Chile, Santiago, Chile; h Cellular Biotechnology Unit, Instituto de Investigación de Enfermedades Raras (IIER), Instituto de Salud Carlos III, ISCIII, Madrid, Spain

**Keywords:** Netrin-1, Neogenin-1, integrin-β1 activation, FAK; cell migration, metastasis, neuroblastoma

## Abstract

Neuroblastoma is a highly metastatic tumor that emerges from neural crest cell progenitors. Focal Adhesion Kinase (FAK) is a regulator of cell migration that binds to the receptor Neogenin-1 and is upregulated in neuroblastoma. Here, we show that Netrin-1 ligand binding to Neogenin-1 leads to FAK autophosphorylation and integrin β1 activation in a FAK dependent manner, thus promoting neuroblastoma cell migration. Moreover, Neogenin-1, which was detected in all tumor stages and was required for neuroblastoma cell migration, was found in a complex with integrin β1, FAK, and Netrin-1. Importantly, Neogenin-1 promoted neuroblastoma metastases in an immunodeficient mouse model. Taken together, these data show that Neogenin-1 is a metastasis-promoting protein that associates with FAK, activates integrin β1 and promotes neuroblastoma cell migration.

## Introduction

Neuroblastoma (NB) is the most common extracranial solid tumor of infancy [1]. It derives from neuroblasts of the sympathetic nervous system, and usually arises in the adrenal gland or sympathetic ganglia [1]. More than 50% of diagnosed cases are metastatic, and hence elucidating the mechanisms underlying NB dissemination is of the utmost importance. Increased cell migration is a central feature in the metastatic cascade [2] and the dynamics of cellular adhesions play a fundamental role in this regard [3]. The so-called focal adhesions (FAs) are supramolecular complexes formed upon engagement and activation of integrins, the main receptors of the extracellular matrix (ECM) [4]. Focal Adhesion Kinase (FAK) is a founding component of FAs, as it promotes both the assembly and disassembly of FAs and hence it is considered a master regulator of cell migration via controlling FA dynamics [5]. FAK is a common downstream molecule of growth factor, axonal guidance receptor, and integrin-triggered signals, all of which converge in cell migration, growth, and survival [6]. Since FAK is upregulated in human NB cell lines and clinical samples, it has been suggested that FAK is important for NB progression and metastasis [7] and other malignancies, such as ovarian serous cystadenocarcinoma, breast invasive carcinoma, and colorectal adenocarcinoma [8,9].

Neogenin-1 (NEO1) is a versatile transmembrane receptor that contributes to NB cell migration and metastasis [10] and is also involved in axonal guidance, neuronal differentiation, morphogenesis, and cell death [11]. NEO1 is ubiquitously expressed during embryonic development, particularly in regions with robust cell proliferation, differentiation and migration [12]. NEO1 was described as a homolog of Deleted in Colorectal Cancer (DCC), as these proteins share about 50% amino acid identity and possess the same secondary structure, consisting of an extracellular domain that contains four Immunoglobulin-like loops and six repeated Fibronectin-III (FNIII) type regions, followed by a single transmembrane region and a cytoplasmic tail, containing three domains conserved with DCC, referred to as P1, P2 and P3 [11]. The P3 domain binds to intracellular proteins that dictate the variety of DCC/NEO1-associated responses [13,14]. The DCC/NEO1 receptors act as homodimers or form heterodimers with the UNC5 receptor family, sharing their binding to the Netrin ligands [15]. Netrin-1 (NTN1) is the best characterized Netrin ligand, and has been shown to interact with NEO1, leading to axonal guidance and cell migration, as well as cell-to-cell adhesion and self reneval [11,16]. The binding between NTN1 and NEO1 involves FNIII domains 4 and 5 of NEO1 [17].

Since both NEO1 and NTN1 are expressed during the development of the sympathetic nervous system [18], their signaling may be relevant within the context of NB oncogenesis and progression. Interestingly, NEO1 promotes the autophosphorylation of FAK on tyrosine 397 (Y397) in cortical neurons, and both proteins have been shown to interact in rat brain [13]. Thus, FAK has been proposed as a downstream signaling molecule of NEO1. Since FAK is suggested to activate integrin-β1 [19], and integrin-β1 is implicated in NB progression [20], it can be inferred that all these molecules have a critical role in NB metastasis. In this study, we sought to elucidate the downstream signaling mechanism associated with NEO1-mediated cell migration and metastasis in NB. Specifically, we show that intracellular signaling triggered by the interaction between NTN1 and NEO1 promotes the activation of integrin-β1 via FAK, leading to NB cell migration and, consequently, metastasis.

## Materials and methods

### Ethical approval and consent to participate

Ethics committees from University of Chile and CONICYT/ANID approved this study. General written consent was obtained from all patients enrolled by HNPG (Hospital de Pediatría Dr. Prof. Juan P. Garrahan), at diagnosis.

### Patient samples and analysis of public databases

All human tumor samples used in this study were diagnosed and morphologically typified, through histological analysis at the Anatomopathological Center of HNPG. Public databases of NB gene expression were visualized from R2: Genomics Analysis and Visualization Platform (http://r2.amc.nl) using MegaSampler analysis to evaluate NEO1 and NTN1 expression across databases. The databases used were Hiyama, Lastowska, and Veegsted. Hiyama database comprises 51 NB samples that were resected either from the patients who died of tumor progression or those whose tumor regressed or matured spontaneously. Lastowska database comprises 30 NB samples and these were obtained from patients of all stages. Versteeg database comprises 88 human NB samples. Importantly, NEO1 expression comparing MYCN amplification was analyzed across those databases, using MegaSampler from R2.

### Immunohistochemistry (IHC) and histological analysis

Paraffin-embedded patient samples of NB were deparaffinated and rehydrated as described in [21]. IHC assays were proceeded by incubating the slides with primary antibodies anti-NEO1 (1:50, sc-15,337, Santa Cruz biotechnologies), anti-NTN1 (1:40, AF6419, sheep, R&D systems) and anti-PCNA (1: 100, 13–3900, mouse, Invitrogen) antibodies in 2.5% horse serum (from the Vestatin kit). The biotinylated secondary antibody was incubated for 2 h, and the ABC kit (Vestatin) was used, revealing with the 39-diaminobenzidine (DAB, Roche) substrate. Hematoxylin was used as a counterstain (Vector Laboratories, Burlingame CA). Images were taken at 100X and 400X. PCNA percentage was calculated by two independent observers, by counting the number of cells marked in quadrants and multiplying by the total number of cells present in each sample, quantified by counting hematoxylin- stained cells. 40% was the median obtained for a total of 21 samples. c square and Fisher’s exact test (n < 5 samples) were used as statistical tests.

### Cell culture

The NB cell line, SK-N-SH (ATCC® HTB-11) was cultured in high glucose Dulbecco’s Modified Eagle Medium (DMEM, Gibco) with 5% Fetal Bovine Serum (FBS, Gibco) and supplemented with antibiotics (Penicillin-Streptomycin, 10,000 U/mL, Gibco). The HEK293FT (human embryonic kidney) cells were cultured in DMEM with 10% FBS supplemented with antibiotics.

### Lentiviral transduction and stable shRNAs cell line generation

To knock-down NEO1 (shNEO1), SK-N-SH cells were transduced with lentiviral particles that contained shRNA vectors (pGIPZ backbone); and two different shRNA sequences (Seq.1 and Seq. 7) were selected and used to knock-down this protein. Sequences are available in Supplementary Figure S2c. A scramble sequence (shSCR) was used as a control. These lentiviral particles were generated using HEK293FT cells, with the CaCl_2_ transfection method [22]. HEK 293 T cells were triple transfected with pCMV-VSV-G, p8.91, and pGIPZ-shRNA (Openbiosystems). After 48 h, the conditioned medium (viral supernatants) of these cells was harvested and added in a 1: 1 ratio to the media of SK-N-SH cells. After 48 h, the transduction percentage was measured using tGFP, encoded in pGIPZ and cells were incubated with the selection marker puromycin (3 mg/ml, Sigma) for an additional 48 h period. Stable cell lines were selected and maintained in DMEM with FBS supplemented with puromycin. The knock-down efficiency was measured via Western blot analysis.

### Transwell migration assays

For migration assays, 8 mm-pore Transwell chambers were used (Corning). As an underside cover, 2 mg/ml of Fibronectin (Sigma Aldrich) was used, placed on the membrane 12 h before performing the assay. As a chemotactic stimulus, different concentrations of recombinant human NTN1 (rhNTN1, R & D Systems) were assayed, all dissolved in DMEM without serum. Briefly, 50,000 shNEO1 and shSCR SK-N-SH cells were used, which were placed in the upper chamber; the lower chamber contained various concentrations of rhNTN1 diluted in DMEM. Cells were incubated for 4 h, then fixed and stained with a solution of crystal violet prepared in methanol, as follows: for stock solution, 0.5 g crystal violet was dissolved in 100 ml methanol (100%). This yielded a 0.5% solution. Then, stock solution was diluted 1/5 in 0.15 M NaCl, thereby yielding a 0.1% solution of crystal violet in 20% Methanol 100% crystal violet diluted in methanol in a 1: 5 solution of 0.15 M NaCl. Images of each condition were taken and five fields per condition were counted.

### Protein co-immunoprecipitation (co-IP)

Protein co-IP was performed as indicated in [10] with variations. To evaluate the interaction between NEO1, integrin-β1 and its ligand NTN1, SK-N-SH cells were incubated with rhNTN1 (200 ng/ml) in DMEM without serum for 1 h. In order to evaluate the interaction between NEO1 and FAK, the cells were treated with NTN1 (25 ng/ml) in DMEM without serum for 1 h. Subsequently, cell extracts were prepared in a buffer containing 20 mM Tris, pH 7.4, 150 mM NaCl, 1% NP-40, and protease inhibitors and incubated by 5 min on ice. The samples were centrifuged at 13,000 g for 5 min at 4°C, and the supernatants (1000 μg of total protein) were incubated with 2 μg of anti-NEO1 antibodies (# sc-6536, Santa Cruz Biotechnology), anti-NTN1 (AF6419, RYD Systems), total anti-FAK (# 05–537, Millipore) or anti-integrin β1 (# sc-8978, Santa Cruz Biotechnology) and immunoprecipitated with Dynabeads protein A (Thermofisher) bead-immobilized antibodies for 1 h. Immunoprecipitated samples were solubilized in loading buffer with ß-mercaptoethanol, and analyzed by Western blot.

### Western blot (WB)

Protein extraction was performed using lysis buffer (SDS 2% w/v, Tris-HCl 80 mM pH 7.5, Glycine 10% w/v) with protease inhibitors (Thermofisher). After three minutes of sonication on ice, samples were centrifuged (10,000 xg) for 5 minutes at 4°C. Samples were resolved in 8% polyacrylamide gels, and proteins were transferred to 0.45 μm nitrocellulose membranes by wet transfer overnight. Primary antibodies were incubated overnight at 4°C in 5% nonfat milk (except for pFAK and NTN1, which is 5% BSA in TBS- 0.01% Tween or RYD systems Buffer 8) diluted in TBS-Tween 0,01%, and the secondary antibodies were incubated at room temperature for 2 h in the same buffer. The antibodies used were anti-NEO1 (# sc-6536, Santa Cruz Biotechnology, 1: 200), anti-NTN1 (AF6419, RYD Systems, 1: 400), anti-actin (A5316, Sigma, 1: 1000), anti-tubulin (T9026, Sigma 1: 1000), anti-pFAK (# 44–624 G, Thermofisher, 1: 1000), total anti-FAK (# 05–537, Millipore 1: 1000), anti-integrin b1 (# sc-8978, Santa Cruz Biotechnology, 1: 300). Western blots were quantified using integrated density analysis with ImageJ software (National Institutes of Health, USA).

### Spreading assay for FAK phosphorylation analysis

SK-N-SH cells were seeded on plates pre-coated with rhNTN1 (2 μg/ml, RYD Systems) or treated with vehicle (PBS) as control for different time periods (0, 15, 30 and 60 min) and proteins were extracted for evaluating FAK phosphorylated on Y397 (# 44–624 G, Thermofisher) and total anti-FAK (# 05–537, Millipore) via WB. Actin (A5316, Sigma, 1: 1000) was used as an internal control.

### Spreading assays and active integrin β1 analysis by immunofluorescence

shSCR and shNEO1 SK-N-SH cells were seeded on coverslips pre-coated with Fibronectin (2 μg/ml, Sigma-Aldrich) for 1 h. Then, the cells were fixed with PFA 4% w/v, stained with phalloidin-546 (Thermofisher) and DAPI. Cell spreading was analyzed by confocal microscopy (Zeiss 710). To evaluate the activation of integrin-β1 [23], pEGFP-NEO1 or the empty vector (pEGFP) were overexpressed and a spreading assay was performed. Briefly, NEO1 was overexpressed in SK-N-SH cells, by using Turbofect (Thermofisher) as a transfection agent. To this end, a pEGFP plasmid containing the complete NEO1 DNA sequence (full length) coupled to eGFP was used. As an empty vector control (EV), the pEGFP plasmid was used. The transfections were performed according to the manufacturer’s instructions and expression of GFP was evaluated at 36 h after transfection by epifluorescence microscopy. Next, cells were seeded on coverslips pre-coated with Fibronectin (2 μg/ml) for 1 h, in DMEM containing rhNTN1 (25 ng/ml). Previously, cells were treated with either the FAK autophosphorylation inhibitor (PF271, TOCRIS) or vehicle alone (DMSO), for 1 h before spreading and the same stimuli were maintained during this test. Then, the cells were fixed with PFA 4% w/v in PBS and the immunofluorescence of activated integrin-β1 (clone Huts-4, MAB2079Z, EMD Millipore, 1: 300) and total integrin-β1 (# sc-8978, Santa Cruz Biotechnology, 1: 100) was performed. The assay was evaluated by confocal microscopy (Zeiss 710) and 400X images were taken. The GFP fluorescence channel was used to select for transfected cells and the fluorescence intensity of the cell periphery was quantified according to the parameters given by the ImageJ software (https://imagej.nih.gov/ij/). For quantification, a 2–3 μm cell perimeter was considered, using Phalloidin staining as a reference for the cell body.

### Spheroid formation and migration assay based on spheroids

SK-N-SH shSCR and shNEO1 were used to perform this assay. Spheroids were generated from 1000 cells seeded in a nonadherent T25 bottle (Corning), with DMEM-F12 and B27 (Thermofisher, 1:50), for 5 days. Once the spheroids were formed, they were harvested and seeded on plates covered with Fibronectin (2 μg/ml) in the presence of DMEM 5% SFB. After 12 h, they were stained with phalloidin-546 (Thermofisher) and DAPI and observed by confocal microscopy (Zeiss 710) counting the cells that migrated away from the spheroids. The spheroids counted were at least 10 per condition per N.

### Spontaneous metastasis assays

Ethical committees from Universidad de Chile, Instituto de Salud Carlos III, and CONICYT/ANID approved animal use and care in this study. shSCR or shNEO1 SK-N-SH cells, stably transduced and subsequently transduced with a plasmid coding for the enzyme luciferase, were used to evaluate metastasis to different organs by luminescence. Briefly, 1.5 million shSCR or shNEO1 cells, mixed with matrigel (1: 5), were injected into both flanks of male immunodeficient mice (NOD SCID gamma, NSG strain. Animals were randomly distributed in both groups, but confounders were not controlled. Based on our previous experience we calculated that 5 animals (10 tumors) were necessary to reach significance in the study. We decided to exclude any animal that would lose more than 20% of weight during tumor growth, but no animal was excluded from the analysis. AAV and PSG conducted the animal´s experiments and the posterior analysis. One week after the injection, the tumor growth was quantified revealing the luminescent activity of the primary tumors, with the use of luciferin (12.5 mg by intraperitoneal injection) in mice, anesthetized with isoflurane. The luminescence was recorded with the IVIS in vivo imaging system (Perkin Elmer), every 2–3 days for several weeks. Animals were killed by cervical dislocation and isofluorane was used to anesthesize the animals for the IVIS analysis. After 5 weeks post-injection, the mice were treated with intraperitoneal luciferin, sacrificed, and the primary tumor was extracted in addition to the following organs: liver, lung, spleen, and kidney. All tissues were analyzed with IVIS equipment and the presence or absence of metastasis was determined for each organ in the different conditions (shSCR or shNEO1). None of the animals or the tumors were excluded from the study. With the extracted primary tumor, and RNA extraction was performed and NEO1 transcript levels were determined to determine if the knock-down remained stable during the 5 weeks procedure.

### Statistical analysis

Values are mean ± S.E.M., where n indicates the number of independent cell cultures. Comparisons between two groups were performed by the Mann Whitney test and unpaired t-test, depending on the parametricity of data and, comparisons between three or more groups were performed via analysis of variance (ANOVA). If the ANOVA demonstrated a significant interaction between variables, post hoc analyses were performed by multiple-comparison Dunn’s correction test. All the determinations were carried out in triplicate. The software GraphPad Prism 5.0b (GraphPad Software Inc) was used for data analysis. p < 0.05 was considered statistically significant.

## Results

### NEO1 is expressed in NB patient samples

**Figure 1.**
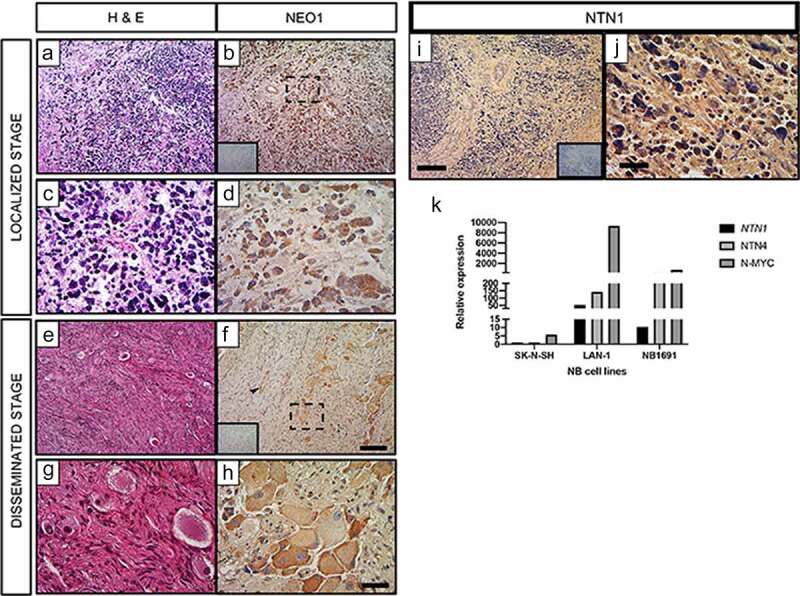
**NEO1 is expressed in NB samples independently of tumoral stage and NTN1 has impaired expression**. Immunohistochemically (IHC) analysis of NEO1 expression in NB samples. In all IHC Hematoxylin was used as a counterstaining a- d: Representative images of a NB patient sample classified at Localized Stage according to INRGSS. a, c: Hematoxylin-Eosin (H&E) staining, b: NEO1 expression (brown). Dotted square shows the area represented at higher magnification in d. e- h: Representative images of a NB patient sample classified at Disseminated Stage according to INRGSS. e, g: H&E staining, f, h: NEO1 expression. Dotted square shows the area represented in high magnification in h. Negative control of the antibody are shown as inset in b and f. Arrowhead indicates NEO1 staining in blood vessels. a, b, e, f: Low magnification Bar: 100 μm, c, d, g, h: High magnification Bar: 20 μm. Immunohistochemistry of NTN1 expression within NB. Representative light microscopy images of neuroblastoma samples from primary tumors. Hematoxylin was used for counterstaining. i, j: Representative images of a NB patient sample classified at Localized Stage according to INRGSS. i: Low magnification, j: High magnification. Negative control of each antibody is shown as an inset. Low magnification Bar: 100 μm, High magnification Bar: 20 μm. k: Relative expression of *NTN1, NTN4* and *N-MYC* in indicated NB cell lines. *GAPDH* expression was used as housekeeping control. N = 21

To determine the contribution of NEO1 in NB progression, we first evaluated its expression in NB patients (n = 21) by immunohistochemistry on paraffin-embedded samples, categorized according to INRGSS (International Neuroblastoma Risk Group staging system) [24]. INRGSS classified the tumors in a pretreatment risk classification system, considering tumor spread and surgical risk factors known as Image Defined Risk Factors (IDRFs) at the moment of diagnosis of the disease [24]. Then, the tumors are classified as Localized Stages (L1 and L2, based on the absence or presence of one or more of 20 IDRFs, respectively), when they are confined in a region, or Disseminated Stages (M, MS), when they are metastatic and aggressive. MS are different from M tumors because metastases are confined to the skin, liver, and/or bone marrow, and they occur in children younger than age 18 months [24]. Figure 1 a-d  shows the expression of NEO1 in a patient sample corresponding to a localized stage (L1) (b, low magnification and d, high magnification). The staining is mostly restricted to tumor cells (Figure 1(b,f), but it can also be found in blood vessels (arrowhead in figure 1f). At disseminated stage (M), as shown in Figure 1(e-h) (f, low magnification and h, high magnification), NEO1 expression persisted in tumor cells. No significant correlations were found between the percentage of NEO1 positive cells and the clinical features, such as gender, age, tumor stage, PCNA expression, primary tumor site, or patient status (Table 1).

**Table 1.** **Characterization of % of NEO1 positive Samples (within tumor cells, blood vessels or stroma)**. Percentage of NEO1 positive samples according to specific clinical characteristics of the patients. 21 patient samples were analyzed and in some cases information were incomplete. We do not found association between percentage of NEO expression and clinical features. Asterisk for p value from Fisher’s exact test

Clinical Feature		% of NEO1 positive Samples	N	χ2	df	p-value	Fisher’s p-value
Gender	Male	78.0	7/9	0.112	1	0.737	1
	Female	77.8	10/12				
Age	>18 M	83.3	8/10	0.015	1	0.9	1
	<18 M	81.8	9/11				
Tumor Stage	Disseminated (M, MS)	80.0	12/15	0.03	1	0.86	1
	Localized (L1, L2)	83.3	5/6				
PCNA	>40%	90.0	9/10	1.014	1	0.314	0.586
	<40%	72.7	8/11				
Primary Tumor Sites	Cervical	66.7	2/3	0.938	4	0.919	1
	Thoracic	100	2/2				
	Abdominal	83.3	5/6				
	Retroperitoneal	80.0	4/5				
	Adrenal	75.0	3/4				
Patient Status	Dead	75.0	3/4	0.257	1	0.612	1
	Recovered	85.7	12/14

Analysis of NEO1 expression across public NB databases using MegaSampler from R2: Genomics Analysis and Visualization Platform (http://r2.amc.nl), revealed that NEO1 expression is similar in the different databases (Supplementary Figure S1a). Details of each database are provided in the Materials and Methods section. Interestingly, when the NEO1 expression data was sorted by MYCN amplification in each database (Supplementary Figure S1b), samples without this amplification showed higher NEO1 expression than MYCN-amplified samples (p value <0.05). Collectively, our data show that NEO1 is expressed in NB patient samples, mostly in tumor cells, and persists throughout different NB stages.

### NEO1 is required for NTN1-induced cell migration

Having shown that NEO1 is persistently expressed in NB samples, we next sought to address the function of NEO1, by shRNA-mediated knockdown in the SK-N-SH NB cell model (MYCN WT), which express higher levels of this gene compared to other NB cell lines [10]. Moreover, these cells are representative of our observations made in other NB cell lines, including LAN-1 and NB1691 [10]. Two different shRNA sequences (Seq.1 and Seq. 7) were used, however, only Seq. 7 substantially decreased NEO1 expression (Supplementary Figure S2 a, Supplementary figure S6f), and hence this shRNA sequence was used for subsequent experiments.

**Figure 2.**
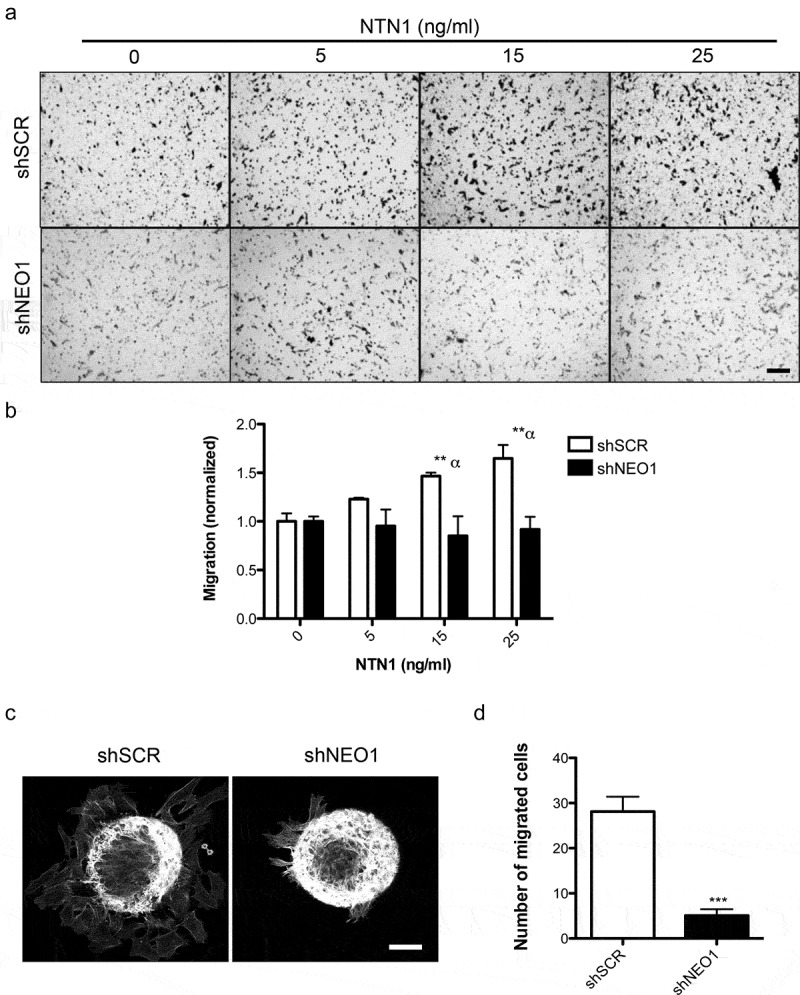
**NEO1 promotes chemotactic NTN1-mediated cell migration**. a: Representative transwell assay images performed with shSCR and shNEO1 SK-N-SH cells which migrated for 4 hours in increasing concentrations of NTN1 indicated in Figure. Bar = 100 µm. b: Quantification of the photographs taken for each condition. Values are expressed as induction times of migration relative to the condition without chemotactic stimulus (0 ng/ml NTN1) for shSCR and shNeo1 cells. N = 3, n = 5 fields per condition were counted, * p < 0.05 0 v/s 25 ng/ml NTN1. c: Representative images of confocal microscopy of spheroid-based migration assay on fibronectin for 1 h, comparing shSCR versus NEO1 knock-down cells. The images reveal F-actin labeling. d: Quantification of cells that migrated away from the spheroid for each condition tested. N = 3, n = 15. *** p < 0.01 shSCR versus shNEO1

Since NEO1 was previously shown to promote NB cell migration [10], we evaluated chemotactic migration of SK-N-SH cells exposed to different concentrations of rhNTN1. Netrins are known to act as chemotactic molecules [25] and NTN1 is the main Netrin ligand of NEO1 and expressed in NB [11]. Indeed, by analyzing the expression of this protein in NB samples we found strong expression in stroma and vessels and, to a less extent, in tumor cells, indicating both autocrine and paracrine NTN1 expression in the tumor microenvironment (Figure 1(i, j). In agreement with our previous results [10], SK-N-SH cells barely expressed endogenous NTN1 (Figure 1k). We speculated that this may represent an informative model to study the paracrine effects of the ligand. Hence, we performed transwell assays with both shSCR (control) and shNEO1 cells, using different concentrations of rhNTN1 (5, 15, 25 ng/ml) in the bottom chamber, allowing cell migration for 4 h. Figure 2a shows representative images of transwell assays and the quantification of these experiments is shown in Figure 2b, indicating that 15 and 25 ng/ml of rhNTN1 increased cell migration in shSCR, but not shNEO1 cells. To confirm the contribution of NEO1 in SK-N-SH cell migration, we made a spheroid-based migration assay. To this end, spheroids formed by shSCR and shNEO1 cells were placed into Fibronectin-coated plates and allowed to migrate for 12 h, fixed, and stained with phalloidin (Figure 2c) to allow quantification of cell migration away from the spheroids. We observed decreased migration of shNEO1 compared with shSCR cells (Figure 2d). Altogether our results indicate that NEO1 is required for NTN1-induced migration in SK-N-SH cells.

### NTN1 induces FAK autophosphorylation and NEO1 binds FAK

**Figure 3.**
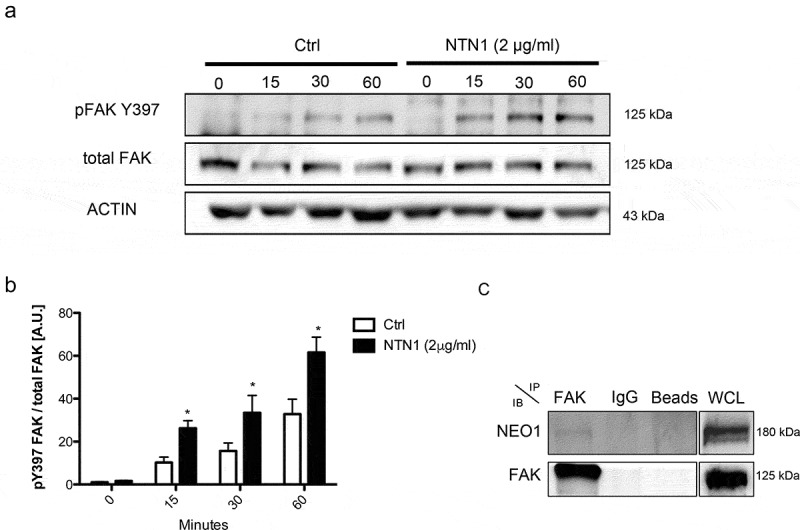
**NTN1 induces FAK phosphorylation and NEO1 binds it**. a: WB of pFAK Y397 and FAK total in cells spreaded onto PBS buffer (Control) and NTN1 (2 μg/ml) at different time periods. Actin was used as an internal control. b: Quantification of WB indicated in a. The ratio between pFAK Y397 versus FAK total was calculated. N = 3, n = 3. c: WB of protein co-immunoprecipitation; FAK was immunoprecipitated and NEO1 was evaluated. WCL: whole cell lysate. N = 3, n = 2

FAK is activated by numerous stimuli, including integrin engagement and growth factor signaling, which converge in cell migration [26]. Hence, we aimed to characterize the potential contribution of this protein in NB migration. We first evaluated the effects of NTN1 on FAK activation in SK-N-SH cells, by assessing its autophosphorylation on Y397 upon cell spreading onto surfaces coated with rhNTN1. Spreading assay permitted visualizing differences between time ‘0’ and subsequent time-points, as cells were synchronized when brought in suspension. By using this approach, cell adhesion promoted FAK phosphorylation in a time-dependent manner (Figure 3a, Supplementary Figure S6a). Importantly, the extent and kinetics of FAK phosphorylation was substantially enhanced in NTN1-coated surfaces (Figure 3(a,b)).

Since FAK was previously shown to interact with NEO1 in whole brain lysates [13], we aimed to evaluate the interaction of NEO1 and FAK in SK-N-SH cells by co-immunoprecipitation. Immunoprecipitation of FAK and subsequent blotting against NEO1 revealed that both molecules associate in a complex (Figure 3c, Supplementary Figure S6b), supporting the possibility that NTN1 signals through FAK via NEO1 activation in NB cells.

### NEO1 and NTN1 form a complex with Integrin β1 in SK-N-SH cells

**Figure 4.**
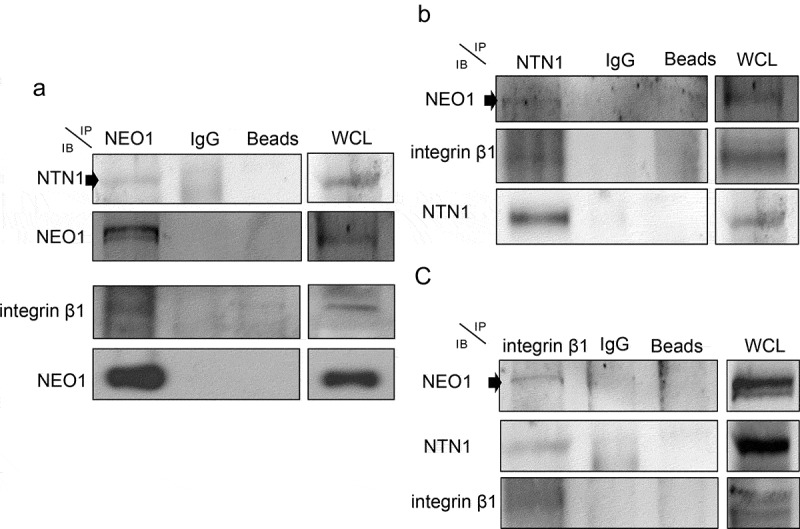
**NEO1/NTN1 form a complex with integrin β1 in SK-N-SH cells**. a: Representative western blots (WB) of protein co-immunoprecipitation assays used to evaluate interaction between NEO1 with NTN1 and integrin β1. b: Representative WB of protein co-immunoprecipitation assays used to evaluate interaction between NTN1 with NEO1 and integrin β1, c: Representative WB of protein co-immunoprecipitation assays used to evaluate interaction between integrin β1 with NTN1 and NEO1. N = 2. WCL: whole cell lysate. N = 2, n = 3

To determine the mechanism underlying NTN1/NEO1-dependent cell migration, we evaluated if these proteins form a complex with integrin-β1 and therefore, a supramolecular structure. NTN1 is known to associate with integrin-β1 in interneurons, promoting cell migration [27], although it cannot be excluded from the possibility that NEO1 is involved in this process. To this end, the association between NEO1, NTN1, and integrin β1, was assessed in co-immunoprecipitation assays in SK-N-SH cells, upon treatment with rhNTN1 (100 ng/ml) for 1 h. Immunoprecipitations were made for NEO1 (Figure 4 a, Supplementary Figure S6c), NTN1 (Figure 4b, Supplementary Figure S6d), and integrin β1 (Figure 4c, Supplementary Figure S6e), and data showed that NEO1 associated with NTN1 and integrin β1. Furthermore, NTN1 was found associated with NEO1 and integrin β1, whereas integrin β1 was also found associated with NTN1 and NEO1. Collectively, these results allow us to suggest the existence of a complex containing NEO1, NTN1, and integrin β1, which may have relevance in NB cell migration.

### NEO1/NTN1 induces integrin-β1 activation via phosphorylation of FAK

Since NEO1 and NTN1 associate with integrin β1, and FAK is a downstream molecule of NEO1 signaling, we further evaluated how these components are functionally related enabling NB cells to respond to external stimuli in a coordinated manner. To this end, we performed co-immunoprecipitation assays, which showed that FAK and integrin β1 associate in SK-N-SH cells (Supplementary Figure S3 a, Supplementary Figure S6h). It has been reported that FAK induces the activation of integrin β1 in human fibroblasts [19] and stimulates cell migration [28]. To evaluate this possibility in the context of NB, we performed a spreading assay in SK-N-SH cells plated on fibronectin for 1 h, and then fixed and labeled against active integrin β1, using a conformational HUTS-4 antibody [29], as was shown in [23]. For quantification, confocal microscopy images were captured at the lower Z-axis of cells undergoing spreading. Subsequently, the fluorescence intensity was quantified for both active and total integrin β1, in the immediate 3 μm layer inside the cell periphery (without considering the nuclear mark). Total integrin β1 immunofluorescence was suited as control. We compared spreading capacity in FAK inhibited cells with 1 mM of PF562,271 [30] (PF271, pFAK Y397 inhibitor efficiency shown in Supplementary Figure S2b, Supplementary Figure S6g) and DMSO control-treated cells (Supplementary Figure S3b). Data are shown as the ratio active/total integrin β1 for each condition and revealed that the inhibition of pFAK Y397 reduced integrin β1 activation (Supplementary Figure S3 c). These results confirm that FAK promotes integrin β1 activation in NB cells.

**Figure 5.**
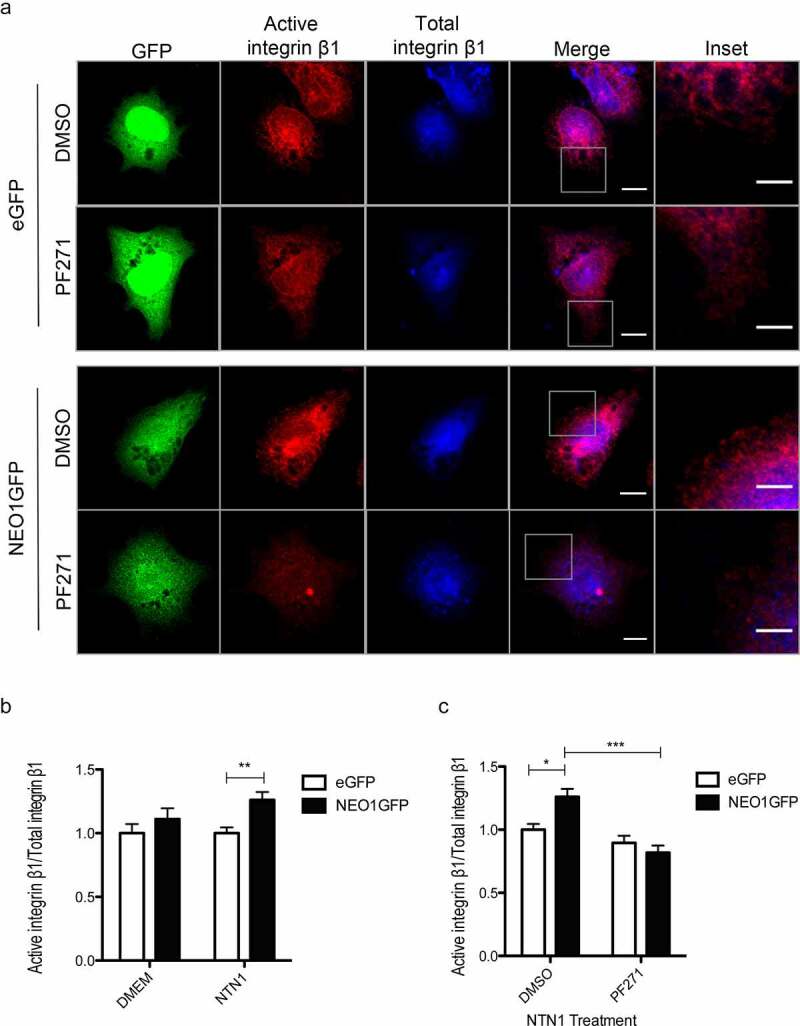
**The NEO1/NTN1 complex induces integrin β1 activation via pFAK**. a: Representative confocal microscopy images of a spreading assay in NEO1 overexpressing SK-N-SH cells (NEO1GFP) versus control eGFP cells in presence of PF271 or vehicle control (DMSO). Immunofluorescence was made using activate integrin β1 (red) and total integrin β1 (blue) antibodies along with transgenic expression of eGFP (green) evaluation. The photos were taken at 400x and the inserts correspond to areas used for quantification Bar: 10 μm. b, c: Quantification of fluorescence intensity between the different conditions for active integrin β1 in relation to total integrin β1 in GFP + cells. Quantification considered the cell edge (2–3 μm) labeled by the F-actin marker. Quantification of the activation of integrin β1 according to NTN1 stimulation (b) and PF271 treatment in NTN1 treated cells (c). N = 3, * p < 0.05. n ≥ 30 cells per condition

Considering that NEO1/NTN1 is associated with integrin β1, FAK is required for the induction of migration mediated by NEO1, and that FAK activates integrin β1, we next evaluated whether NEO1 promotes the activation of integrin β1 through FAK autophosphorylation. NEO1 is a dependence receptor [10] and hence its overexpression in a long term induces cell death. Thus, we overexpressed NEO1 in SK-N-SH cells at low concentrations (WB in Supplementary Figure S4, Supplementary Figure S6i) and performed a spreading assay using the same conditions as previously reported, in presence of the FAK inhibitor PF271, upon stimulation with rhNTN1 (25 ng/ml). Figure 5a shows representative confocal images of the assay. Quantification in Figure 5b shows that NTN1 significantly increased the activity of integrin β1 in NEO1-overexpressing cells, indicating that NTN1 is required for NEO1 to induce the activation of integrin β1. However, treatment with PF271 prevented NTN-induced activation of integrin β1 in NEO1-overexpressing cells (Figure 5c). The latter indicates that FAK autophosphorylation is required for the induction of integrin β1 activation downstream of NTN1 activated NEO1 signaling.

### *NEO1 promotes metastasis* in vivo

**Figure 6.**
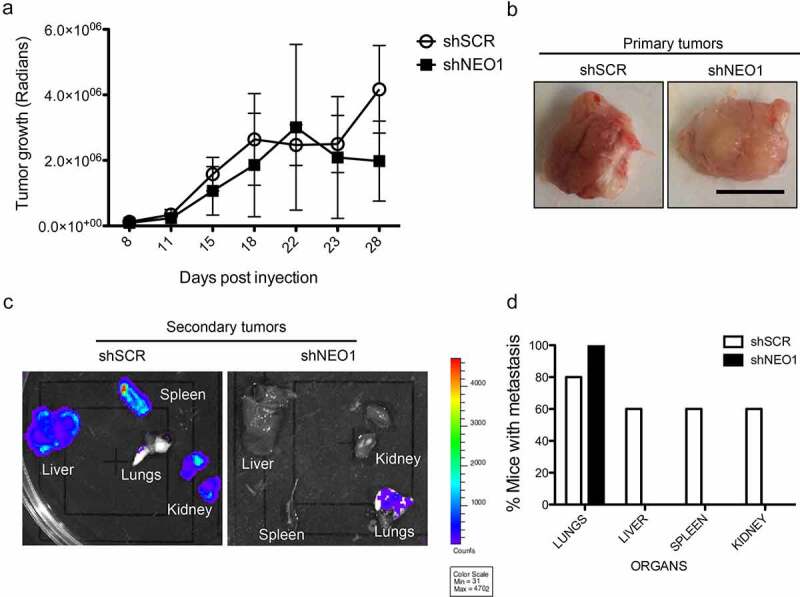
**NEO1 promotes metastasis *in vivo***. Stable luminescent shSCR (Control) and shNEO1 cells were injected in flank of NSG mice (n = 5 for each group). After 5 weeks, primary tumor and several organs were extracted and analyzed using IVIS Ilumina III *in vivo* imaging system. a: Tumor growth of shSCR and shNEO1 primary tumors (n = 10 for each group). b: Representative images of primary tumor for each condition. Bar: 1 cm. c: Representative images of organs visualized in IVIS. d: Graphic representation of metastasis results. Five specimens per injected cell type were analyzed. Presence or absence of metastasis in each organ was scored. Percentages of metastasis were indicated for each cell type in each organ. N = 5, n = 2

After determining that NEO1 promotes NB cell migration *in vitro* and having established a possible mechanism associated with this process, we decided to evaluate the role of NEO1 in an *in vivo* model of metastasis. Accordingly, immunodeficient mice (NSG strain) were injected in both flanks with NEO1 knock-down cells (shNEO1) or control cells (shSCR). Figure 6a shows a comparison of the growth curve for shSCR versus shNEO1 SK-N-SH-derived primary tumors, indicating no significant differences in tumor growth. Moreover, Figure 6b shows a similar size of representative primary tumors at the endpoint. To determine that silencing of NEO1 was not lost during the assay, receptor mRNA levels were measured in the primary tumors for both conditions verifying that silencing is stable and maintained in an *in vivo* context (Supplementary Figure S5). Five weeks after implanting the primary tumors, spontaneous metastases were evaluated in different organs (lung, liver, kidney, spleen). In Figure 6c, secondary tumors are shown in different organs. It is noteworthy mentioning that we found practically no metastasis in the animals where shNEO1 cells were implanted, except in the lung. However, shSCR cells generated metastasis in all the organs analyzed (Figure 6d). The individual data of each mouse analyzed, and the qualitative luminescence intensity is depicted in Supplementary Table S1. These results suggest that NEO1 is required for NB metastasis *in vivo*.

## Discussion

NB is a pediatric tumor arising from an embryonic sympathoadrenal lineage of the neural crest [31] and the first cause of death from pediatric cancer for children under five years. NB is a very aggressive tumor, where almost 50% of cases diagnosed are metastatic [32]. However, the mechanisms behind this process are still not well described.

NEO1 is a multifunctional receptor that has been involved in differentiation, cell death, angiogenesis, axonal guidance, and, in the last few years, cell self-renewal and migration, in the context of development [33]. NEO1 is also relevant for cell migration in several cancers, including gastric cancer [34] and NB [10], however, the associated signaling mechanisms have not been elucidated. The aim of this work was to evaluate how NEO1 induces chemotactic cell migration through its ligand, NTN1, and to evaluate whether their signaling contributes to NB metastasis. This work demonstrated for the first time that the association of NEO1 with NTN1 induces FAK phosphorylation and consequently the activation of integrin β1, in a FAK-dependent manner. These observations provide novel insights into the mechanism whereby NEO1 induces NB cell migration and metastasis. Although it could be argued that other NEO1 ligands may be involved in such mechanism, we could envision that these events are rather specific for NTN1 acting as a chemoattractant being expressed mainly in the neighboring stromal cells. Of note, NEO1 was found to be the most predominant NTN1 receptor since RGMA and DCC are not expressed in NB cells explored herein [10,35]. Taken together, our data support the view that NTN1 and its NEO1 receptor are upregulated in the NB tumor niche, triggering metastasis.

### Clinical significance of NEO1 expression in NB patient samples

A previous report from our group [10], showed that NEO1 expression in NB public database is correlated with a low survival rate, indicating a possible role of NEO1 in the pathogenesis of this cancer. The analysis of NEO1 expression across diverse datasets revealed that it is stable at different NB stages, which correlates with the IHC analysis performed in our cohort of samples. Furthermore, NEO1 expression is mostly restricted to tumor cells and is persistent in all tumoral stages analyzed. Thus, this elevated NEO1 level suggests a selective advantage acquired by cancers cell to migrate and metastasize. To date, the amplification of MYCN remains the best-characterized genetic marker of risk in NB. Aberrant expression of MYCN has been associated with tumor aggressiveness, resistance to chemotherapy, and the inability to differentiate [36]. Interestingly, when NEO1 expression was evaluated in different NB datasets, we observed that it is preferentially expressed in patient samples without MYCN amplification. Noteworthy, integrin β1 also shows a negative correlation with MYCN amplification in NB [37]. Hence, a differential mechanism of cell migration, active in MYCN amplificated tumors could be proposed, a matter that requires further research. Since both NEO1 and integrin β1 are more expressed in patient samples without MYCN amplification and both are important for NB cell migration, we considered interesting evaluating whether there is a functional relationship between these proteins in the process of cell migration and metastasis in the well characterized SK-N-SH (non-MYCN-amplified) cells.

NEO1 has several ligands, including NTN1, which is the best-characterized member of the NTN laminin-related group composed by NTNs 1–4. Nowadays, there is a growing collection of information regarding the different biological roles that NTN1 displays in a variety of cancer types [38,39]. However, the signaling pathway that is activated downstream of NTN1 is an issue that remains to be resolved [40]. In our analysis, NTN1 is expressed in the NB microenvironment. NTN1 is being secreted by the stroma and vessels of the NB primary tumors, corroborating prior literature pointing to an expression in lung and colon tumor stromal cells [39] . NTN1, being a secreted factor, is present in multiple tissues. It has been found outside the central nervous system in the blood plasma [41] and urine [42] as well as in endothelial cells [25], medulloblastoma [42] and colorectal tumor cells [43], among others. Keeping in mind that NEO1 is a dependence receptor and, as such, requires a ligand to execute a non-apoptotic/positive signaling, we propose that NTN1 promotes cell migration and invasion. Moreover, we have recently shown that NTN4 is also present in the NB niche, being strongly expressed by blood vessels [44]. Therefore, it is relevant to consider the tumor microenvironment, including the tumor stroma, and other elements, such as the endothelial niche, as being essential to sustain tumor growth and metastasis.

### Mechanisms associated to NEO1/NTN1 complex association with integrin β1 and cell migration

Here we show that NEO1 induces cell migration through NTN1 mediated chemotaxis in SK-N-SH cells. This result is concordant with data exposed in 2015 by the O’ Leary group [33] who showed that, in a physiological context, NTN1 induces neuroblast cell migration via its receptor NEO1. Our results are also in line with recent data by Yin et al. [45] revealing that the NTN1/NEO1 signaling pathway plays an important role in gastric cancer progression.

Hence, we aimed to determine how the interaction between the above-mentioned molecules commands downstream signaling in NB. Previously, it has been reported that NTN1 binds integrin β1 [26] regulating the migration of interneurons during development. Nevertheless, the authors did not evaluate a possible NEO1 association with integrins in this process. Here, we show that NEO1 associates with integrin β1 and its ligand NTN1, through co-IP analysis, forming a ternary complex. Our result could reconcile the disparities of NTN1 reported functions according to ECM or concentrations. For example, when studying axon guidance in cultured dorsal root ganglions, NTN1 causes a collapse of growth cones extending on high levels of laminin-111, but not on low levels of laminin-111 or Fibronectin [46]. This differential phenomenon could be explained by the NTN1 concentration used because NTN1 has different functions according to cell type analyzed (e.g. reduces chemotaxis of neutrophils [47]) or concentration (high or low concentrations [25]), binding different receptors such as UNC5 [48], which has a chemorepulsive function. Hence, according to ligands concentration, NTN1 could command different processes, mediating chemoattraction via NEO1 or repulsion through the UNC5 family.

In axonal guidance [13] and muscle development [49], NEO1 induces FAK autophosphorylation in Y397, exposing other p-FAK domains and promoting further FAK activation. In this study, we found not only interaction between NEO1 and FAK but also the induction of FAK Y397 phosphorylation when cells are treated with exogenous NTN1. Importantly, our results are in line with recent data published by Huyghe et al, who indicated that NTN-1 signaling through NEO1 promotes FAK activation in mouse embryonic stem cells [16]. In addition, p-FAK Y397 inhibitor reduces cell migration of control cells to a similar extent when compared to NEO1 knock-down cells in a cell migration assay, indicating that FAK is downstream of the NEO1 signaling pathway. NEO1 interacts with FAK through its intracellular P3 domain, as reported also for its homolog DCC. Recent findings showed that once the NTN1/DCC signaling pathway is activated, binding of the P3 domain of the receptor to the focal adhesion targeting (FAT) domain of FAK is produced, as evaluated through crystallography [50]. Despite the fact that the FAK FAT region is the binding domain to talin and/or paxillin [51], there are different recognition sites in the FAT domain when bound to the DCC P3 domain . Also, this binding recruits FAK close to the cell membrane, which could exert a concerted effect for FAK signaling, including the activation of integrins. Indeed, FAK has been syndicated as an important hub molecule in integrin activation [19] (Supplementary Figure 3 b, c), associated in nascent focal contacts [52], where this signaling is initiated. Moreover, FAK is considered an integrator between receptors and integrin signaling [53]. Whether additional, yet unknown signaling pathways are involved in NTN1/NEO1/FAK/integrin β1 activation, such as RIAM/Rap1/Talin, could not be excluded. Further research is required to assess these possibilities. Also, the requirement of α subunits that associate with β1 remain to be explored, although previous findings indicate that a subset of these subunits, including α2 and α3, are expressed in SK-N-SH cells [54]. Finally, we cannot exclude the possibility that a fraction of NTN1 could be binding to α3/integrin β1.

Having shown that FAK is a downstream molecule of NTN1/NEO1 signaling and having established an association between this complex and integrin β1, we aimed to determine if NEO1 is involved in integrin β1 activation in SK-N-SH cells. As predicted, overexpression of NEO1 in presence of NTN1 treatment-induced integrin β1 activation. Of note, binding of NEO1 with its ligand NTN1 is required to induce integrin β1 activation, since absence of exogenous NTN1, did not result in any significant increment of active integrin β1. Also, pharmacological inhibition of phosphorylation of FAK Y397 reduced levels of active integrin β1 in NEO1 overexpressing cells, indicating that phosphorylation of the kinase is important to promote this activation. Although we provide evidence about phosphorylation of Tyr397, we cannot exclude other downstream phosphorylation events. . Most FA proteins contain multiple binding sites for other proteins; therefore, other supramolecular structures could form within FAK binding sites. Thus, the association between NEO1 and integrin β1 could not only promote the integrin β1 activation but also lead to other FAs protein activation. Clearly, the possible intricate network of the interplay between these proteins warrants further investigation. At this point, we cannot rule out that the turnover of FA’s downstream of FAK might reasonably be altering the trafficking of the integrins (and the formation of complexes with NTN1/NEO1 and/or UNC5) but these are never explored.

In summary, here we propose a mechanism whereby NEO1, in interaction with NTN1, associates with integrin β1 and induces its activation via FAK phosphorylation in SK-N-SH cells. Further studies are required to explore whether this mechanism could be generalized to NEO1 signaling in malignant cells.

### NB metastasis promoted by NEO1

The participation of NEO1 in NB cell migration, together with the fact that NEO1 knock-down cells were less metastatic in a chorioallantoic membrane assay [10], led us to evaluate in this work the potential role of NEO1 in NB metastasis using a immunodeficient mouse model and a spontaneous metastasis approach. Control cells metastasized to the spleen, liver and kidney in 60% of mice analyzed, and to the lungs in an 80% of the cases. Meanwhile, knock-down cells, exclusively metastasized to the lungs (in 100% of the mice analyzed). Lungs are a preferential niche in several cancer metastases [55], as they are very blood irrigated and present an intricate vasculature, promoting extravasation of tumoral cells. Considering that NEO1 knockdown was partial (60% of reduction), we could hypothesize that the remaining NEO1 protein could facilitate lung metastasis. In any case, our results indicate that NEO1 is necessary for NB SK-N-SH cells metastasis in an immunodeficient mice model. NTN1, although expressed by tumor cells, is mostly located either in adjacent endothelial cells or stroma, suggesting a relevant contribution to this pathology acting as chemotactic molecule. Importantly, NTN1 recently is upregulated in cancer-associated fibroblasts, modulating tumor plasticity [39]. Therefore, it is relevant to consider the tumoral/stromal/endothelial niche as being essential to sustain tumor growth and metastasis.

In conclusion, NEO1 binds to its NTN1 ligand, signaling downstream with integrin β1/FAK and promoting metastasis in NB. These findings may be beneficial to the understanding of the cellular mechanisms of NEO1 function. Future studies in preclinical models need to address if this molecular crosstalk is preserved and could represent a possible therapeutic target. Currently, efforts to develop drugs that inhibit the interaction of NTN1 with its receptor are underway; the first clinical trial of a humanized-monoclonal antibody targeting the ligand will be completed in 2022 (www.clinicaltrials.gov, NCT02977195). Our final goal is to translate our results into better therapeutic strategies, through precision medicine, contributing to the diagnosis and treatment of NTN1/NEO1-driven tumors.

## Supplementary Material

Supplemental Material

## Data Availability

All the data are available. Public databases were referentied in the material and methods section.
