# Supplementary figures and images for: The Netrin-1-Neogenin-1 signaling axis controls neuroblastoma cell migration via integrin-β1 and focal adhesion kinase activation
Source: Cell Adh Migr. 2021 Mar 16;15(1):58–73. doi: 10.1080/19336918.2021.1892397 (PMC7971226; doi:10.1080/19336918.2021.1892397)

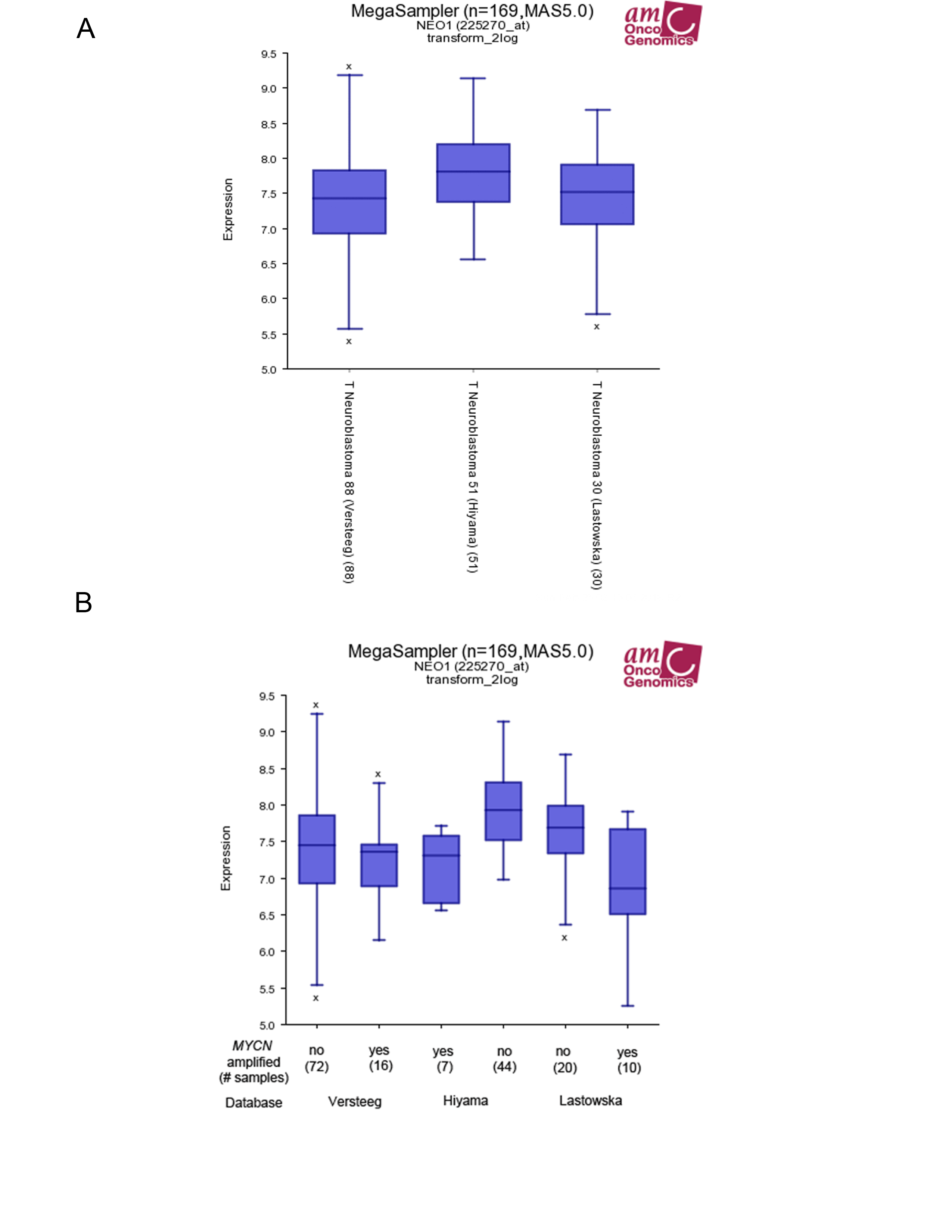

Supplement: Supplemental Material [file KCAM_A_1892397_SM3609.zip › Supplementary information/supp 1.tif]

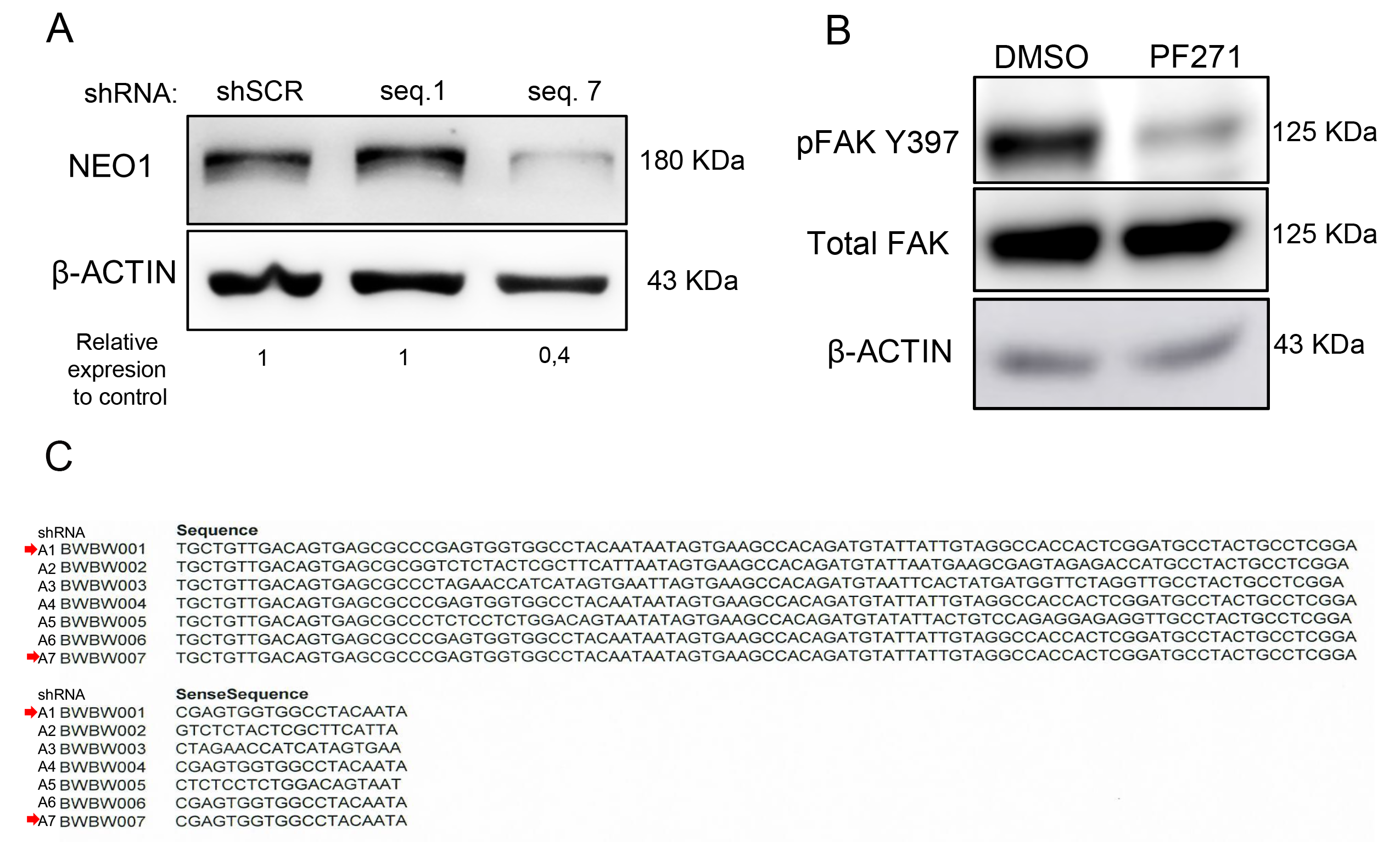

Supplement: Supplemental Material [file KCAM_A_1892397_SM3609.zip › Supplementary information/supp 2.tif]

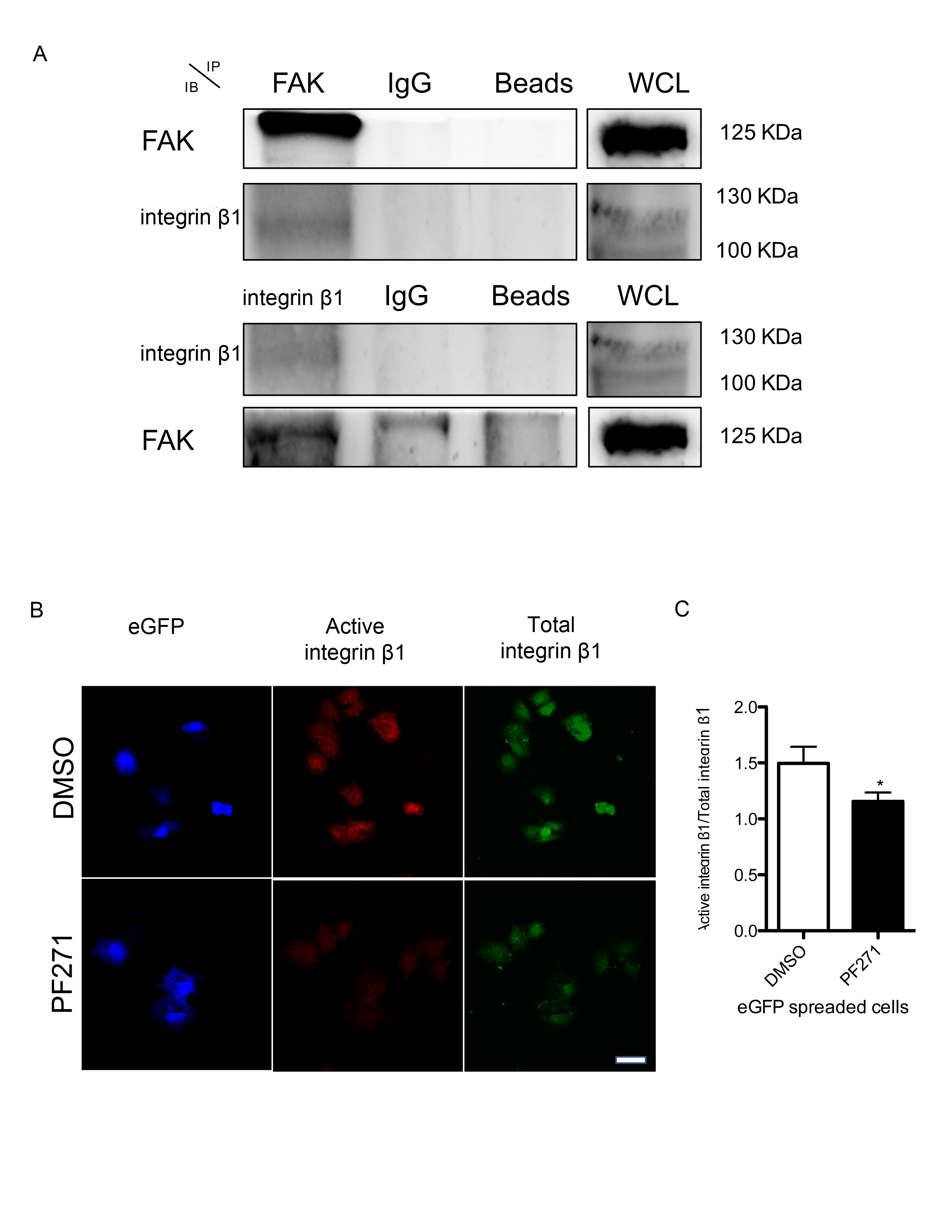

Supplement: Supplemental Material [file KCAM_A_1892397_SM3609.zip › Supplementary information/supp 3.tif]

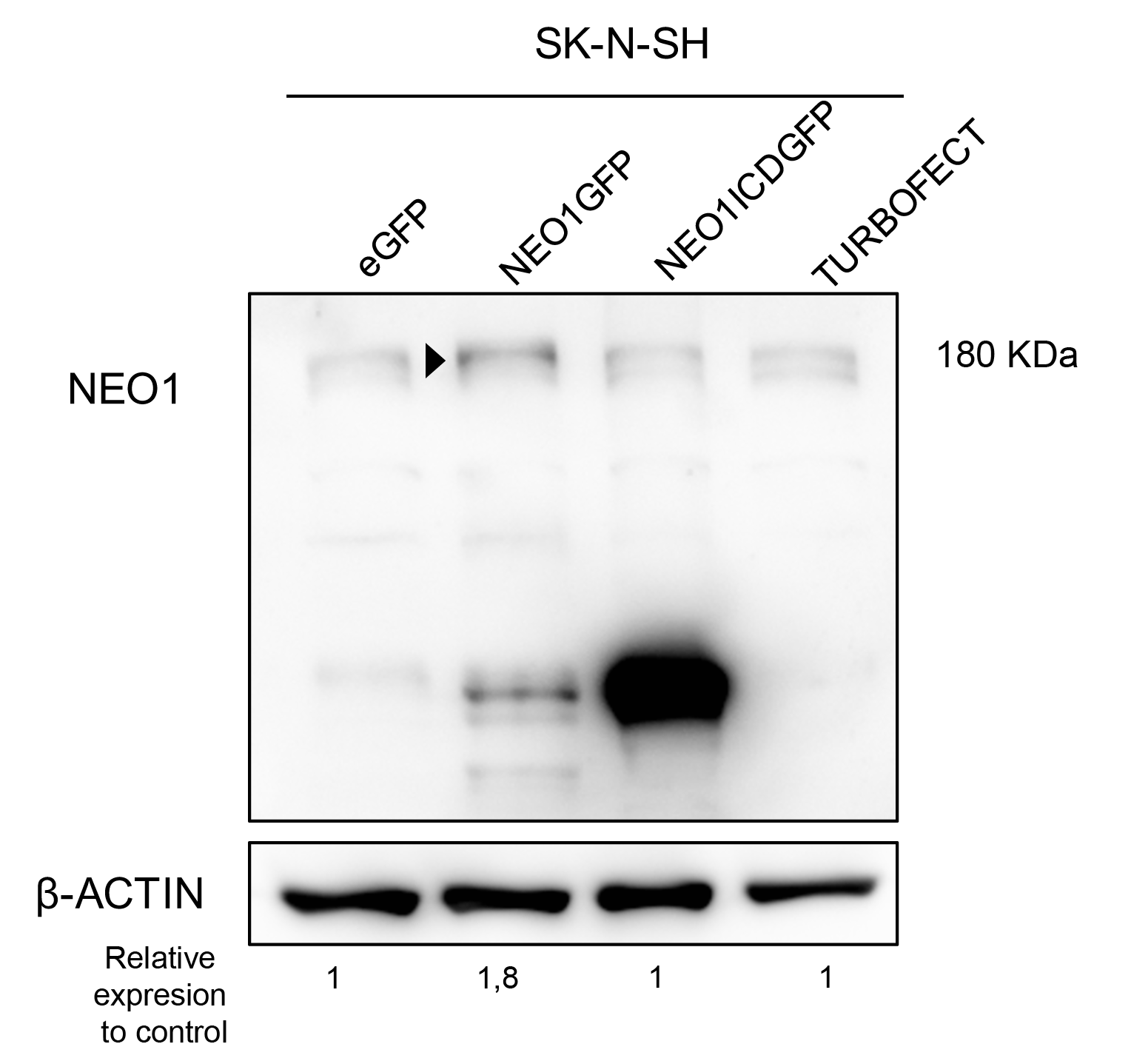

Supplement: Supplemental Material [file KCAM_A_1892397_SM3609.zip › Supplementary information/supp 4.tif]

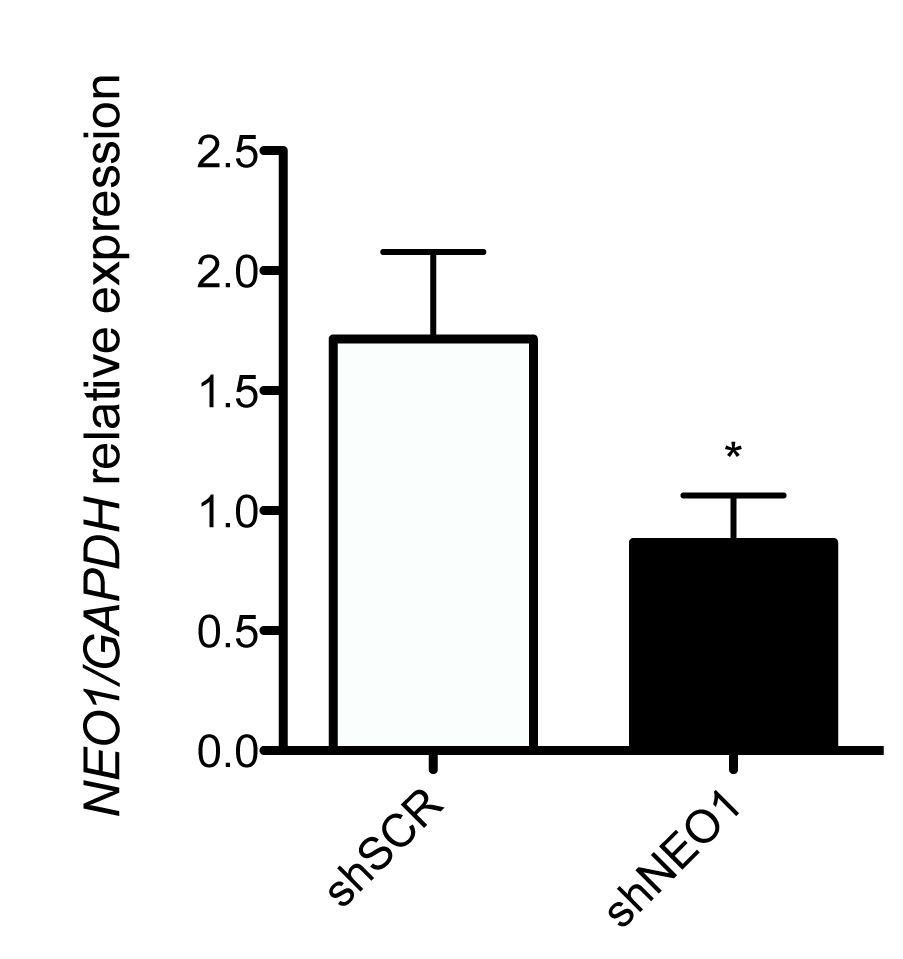

Supplement: Supplemental Material [file KCAM_A_1892397_SM3609.zip › Supplementary information/supp 5.tif]

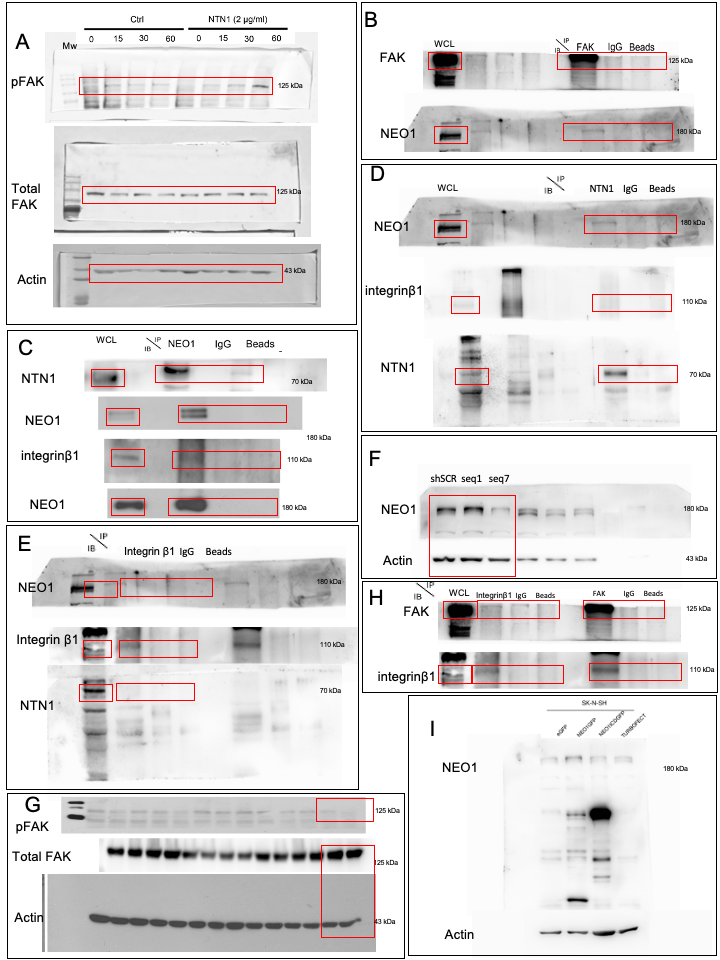

Supplement: Supplemental Material [file KCAM_A_1892397_SM3609.zip › Supplementary information/Suppl fig 6.tiff]
